# Analysis of safety and efficacy of conservative treatment and endovascular treatment in patients with spontaneous isolated mesenteric artery dissection

**DOI:** 10.3389/fsurg.2022.944079

**Published:** 2023-01-06

**Authors:** Xiaobin Chen, Lei Xu, Zhaojun Xu, Zuyou Fan, Jianqiang Huang, Junjie Li, ZaiZhong Zhang, Chen Lin

**Affiliations:** ^1^ Department of General Surgery, Fuzong Clinical Medical College, Fujian Medical University, Fuzhou, China; ^2^ Department of General Surgery, 900th Hospital of Joint Logistics Support Force, Fuzhou, China; ^3^ Graduate School, Qinghai University, Xining, China; ^4^ Department of General Surgery, Dongfang Hospital of Xiamen University, School of Medicine, Xiamen University, Fuzhou, China

**Keywords:** acute isolated mesenteric artery dissection, endovascular treatment, conservative treatment, efficacy, complications

## Abstract

**Background:**

Spontaneous isolated superior mesenteric artery dissection (SISMAD) is a rare disease with abdominal pain as the main clinical manifestation, but its optimal treatment strategy has not yet been determined. Based on this, this study explored a safe and effective treatment method by analyzing and comparing the safety and efficacy of conservative treatment and endovascular treatment in SISMAD patients.

**Methods:**

The clinical and imaging data and treatment effects of 85 patients with SISMAD who were admitted to the General Surgery Department of the 900th Hospital of the Joint Logistics Support Force of the Chinese People's Liberation Army from January 2008 to December 2020 were retrospectively analyzed. Two groups were treated, the data of patients in conservative treatment group and endovascular treatment group were analyzed, and a safe and effective treatment method for SISMAD was discussed.

**Results:**

The mean follow-up time was 36.58 ± 25.03 months. The success rate of interventional operation was 86.11% (31/36), and the operation failed because the guide wire could not enter the true lumen in four cases. One case was terminated due to poor physical condition of the patient who could not tolerate surgery. There were no significant differences in gender, body mass index, clinical manifestations, and past history between conservative treatment and endovascular treatment (*P* > 0.05), but in age, superior mesenteric artery-distal aorta angle, distance from the superior mesenteric artery opening to dissection, dissection length, and true lumen stenosis. There was a statistical difference between the two groups in the rate and Yun classification (*P* < 0.05).

**Conclusions:**

Conservative treatment is effective for most symptomatic SISMAD patients, and close monitoring is required; for patients with persistent symptoms and severe true lumen stenosis (especially Yun classification type III), endovascular treatment is preferred; endovascular treatment is mainly based on endovascular bare stent placement. Patients receiving stent implantation may suffer from stent stenosis or occlusion in the long term, and most of them have no obvious symptoms of intestinal ischemia; the prognosis is good.

## Introduction

Spontaneous isolated superior mesenteric artery dissection (SISMAD) refers to the dissection that occurs only in the superior mesenteric artery (SMA). It has been well known since it was first reported by Bauersfeld in 1947 (1). As a rare vascular disease, the incidence of related autopsy reports is only 0.06% (2). In recent years, with the follow-up and popularization of imaging methods such as CTA and MRA, more and more SISMAD patients have been diagnosed, including clinically asymptomatic patients (3, 4).

As a rare clinical disease, its pathogenesis is still unclear. At present, it is mainly considered that diabetes, hypertension, hyperlipidemia, smoking, atherosclerosis, fibromuscular dysplasia, Behçet’s syndrome, and connective tissue disease are related to its pathogenesis (5, 6). In addition, Karaolanis et al. (5) pointed out that a new hypothesis implicating the transition from a relative fixed to an unfixed arterial segment has been proposed as a cause for dissection. In order to prevent the rupture and hemorrhage of dissecting aneurysm, further progress of dissection leading to intestinal ischemia and necrosis, and to improve the abdominal symptoms of patients, the treatment methods of SISMAD mainly include conservative treatment, endovascular treatment, and open surgery, among which conservative treatment is the main preferred treatment method, mainly for patients with no signs of intestinal ischemia. Endovascular therapy is the preferred method for symptomatic patients (mainly ruptured dissecting aneurysm, intestinal ischemic necrosis, etc.) due to its advantages of less trauma (7). At present, many articles have reported that endovascular stent placement is the preferred treatment for patients with symptomatic SISMAD, mainly including bare stents and covered stents. However, it is still controversial whether covered stent or bare stent is preferred for the treatment of superior mesenteric artery dissection. At the same time, there have been studies on the efficacy and safety of endovascular therapy, but the number of cases is relatively small and there are few related reports in China. Based on this, this study retrospectively analyzed the clinical data of 85 SISMAD patients admitted to our hospital, combined with relevant domestic and foreign literature, and compared the safety and effectiveness of conservative treatment and endovascular treatment in SISMAD patients, so as to provide relevant reference for the treatment of this disease.

## Materials and methods

### Data collection

**Figure 1:**
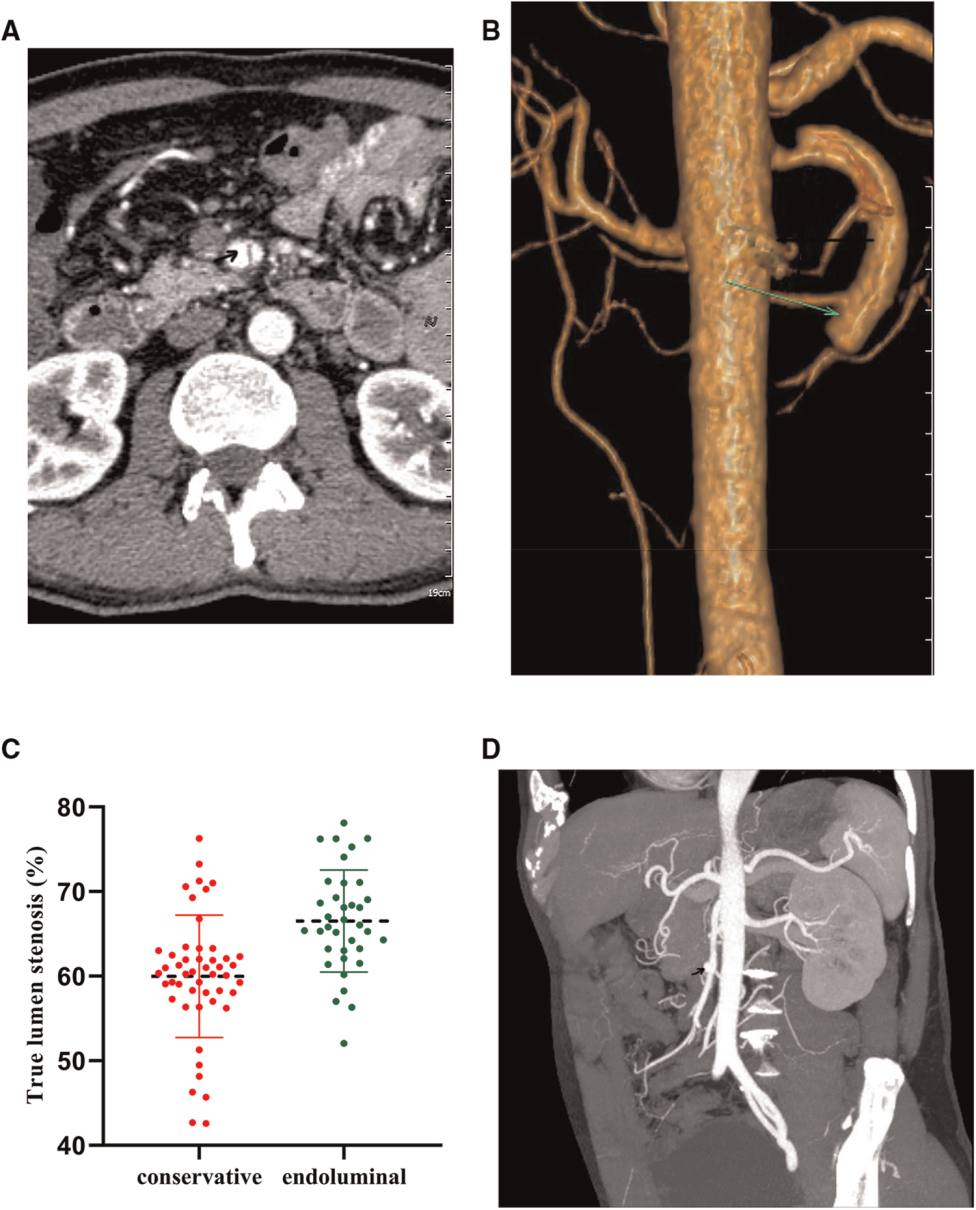
Diagnostic abdominal CTA. (**A,B**) Typical cases of the endoluminal treatment group. (**C,D**) Typical cases of the conservative treatment group.

The clinical and imaging data of 85 patients with ISMAD admitted to the 900th Hospital of the Joint Logistics Support Force of the Chinese People's Liberation Army from January 2008 to December 2020 were retrospectively analyzed. Treatment methods include conservative treatment and interventional endovascular treatment. Inclusion criteria are (1) symptomatic patients; (2) patients with superior mesenteric artery dissection confirmed by CTA without aortic dissection, as shown in Figure 1; (3) patients without rupture of dissecting aneurysm. Exclusion criteria include (1) patients with coeliac artery dissection; (2) allergy to contrast medium; (3) abdominal trauma or surgery history; (4) tortuous abdominal aorta, unable to delineate the long axis of abdominal aorta; (5) patients with incomplete preoperative imaging examination data. In this study, the Yun (8) classification system was used to classify SISMAD into four types: type I, the true and false lumen are unobstructed, and the false lumen has an entrance and an exit; type II, the true lumen is unobstructed, the false lumen has an entrance, and no exit (Type IIa, there is blood flow in the false lumen; type IIb, thrombosis in the false lumen often causes stenosis of the true lumen); type III, dissection and SMA occlusion.

### Indicators included in the analysis

Collect general patient information and imaging results, such as patient age, gender, body mass index (BMI), time from symptom onset to hospital admission, clinical presentation, numerical rating scales (NRS) for abdominal pain before and after surgery, previous history (smoking history, hypertension, diabetes, high fat, blood sugar), imaging manifestations [with or without dissecting aneurysm (refers to SMA dissection with lumen aneurysm-like expansion, more than 1.5 times the diameter of surrounding normal blood vessels)], superior mesenteric artery-distal aorta angle (SAA): CTA examination, all patients were scanned with GE Light-speed64-slice spiral CT before operation and three-phase enhanced scanning. All images were reconstructed at a workstation, and SMA and the largest diameter of abdominal aorta were at the same level on the sagittal multiplanar reconstruction graph. The angle between the anterior wall of the aorta and the inferior wall of the superior mesenteric artery was measured, along with the distance from the opening of the SMA to the beginning of the dissection, and the dissection length, inner diameter of the true lumen of the superior mesenteric artery, true lumen stenosis rate [(the lumen diameter at the proximal end of the dissection-the lumen diameter at the most stenotic point)/the lumen diameter at the proximal end of the dissection × 100%], postoperative abdominal pain, and reexamination CTA situation., etc.

### Treatment mode

#### Conservative treatment

For patients with good general condition, it generally includes strict control of blood pressure, fasting water, gastrointestinal decompression, acid suppression, pain relief, nutritional support, antiplatelet, anticoagulant therapy, etc.; antiplatelet drugs can be aspirin, clopidogrel. or a combination of both. Anticoagulant drugs, including warfarin, low-molecular-weight heparin, rivaroxaban, etc., can be used for treatment in the case of complicated thrombosis. When the patient's abdominal symptoms improved or disappeared, resumed a normal diet, and the reexamination of CTA showed that the dissection did not progress, the patient was discharged. After discharge from the hospital, oral antiplatelet drugs (aspirin enteric-coated tablets 100 mg, q.d.) and drugs to improve circulation (sarpogrelate hydrochloride tablets 100 mg, t.i.d.) were continued for at least half a year.

#### Endovascular treatment

The patient was placed in the supine position, routinely disinfected bilateral groins and right upper limbs, and sterile towels were spread. After successful local anesthesia, the right femoral artery or right brachial artery was punctured by the Seldinger method. First, the F6 catheter sheath was placed into the blood vessel through the puncture point, and the whole body was heparinized (unfractionated heparin, 100 U/kg), and selective SMA angiography was performed through the catheter. The main form of endovascular treatment is stent implantation. Bare metal stents are generally used. The length of the stent should cover the whole course of the lesion as much as possible, and the diameter of the stent should be based on the diameter of the initial segment of the SMA at the proximal end of the lesion. For lesions with severe stenosis or even complete occlusion of the true lumen, balloon dilation + stent implantation is performed (9). When the pressure in the false lumen is larger and the true lumen is partially compressed, the false lumen spring plug is used for embolization on the basis of stent implantation in the true lumen. For severe stenosis or occlusion of the true lumen caused by thrombosis in the true lumen, direct thrombolysis (catheter-directed thrombolysis, CDT) is performed (9). Technical success is defined as restoration of unobstructed blood flow in the true lumen of SMA and no blood flow through the false lumen (3). After the operation, SMA angiography was performed again to clarify the SMA and distal blood flow. After operation, low-molecular-weight heparin anticoagulation (100 U/kg, q12h) was continued to prevent acute stent thrombosis; two drugs combined with antiplatelet therapy were continued outside the hospital (aspirin enteric-coated tablets 100 mg, q.d.; clopidogrel hydrogen sulfate tablets 75 mg, q.d.) for 6 months, and then changed to oral single antiplatelet drug therapy (aspirin enteric-coated tablet 100 mg, q.d.) to 1 year after surgery.

#### Open operation

When there are signs of intestinal ischemic necrosis or rupture of a dissecting aneurysm, open surgery is the first choice. Commonly used open surgical methods include SMA endarterectomy + patch angioplasty, abdominal aorta-SMA bypass, etc. When intestinal necrosis occurs, the necrotic intestinal tube needs to be removed at the same time. After the operation, he was transferred to the intensive care unit for advanced life support treatment, and he was instructed to fast after the operation. After the intestinal function was restored, the diet was gradually restored to promote the recovery of intestinal function.

### Follow-up

All patients were followed up regularly for 1 month after surgery, 3 months after surgery, half a year after surgery, 1 year after surgery, and once a year thereafter. Follow-up was conducted through outpatient clinics, text messages, telephone calls, and WeChat. Follow-up content included the presence or absence of abdominal symptoms, imaging findings of abdominal CTA [vascular remodeling (complete vascular remodeling was defined as complete disappearance of dissection on imaging, no residual stenosis or thrombus in SMA 3)], presence or absence of stent stenosis or thrombus occlusion, etc. The follow-up cut-off time was April 5, 2022, when the patient died or reocclusion occurred. Overall survival was defined as the time from postoperative day 1 to death or the end of follow-up.

### Statistical analysis

SPSS 26.0 software was used for statistical analysis of the data. The normally distributed measurement data were expressed as (x ± s), and the skewed data were expressed as [M(P_25_, P_75_)], and the measurement data met normality and homogeneity of variance. The *t* test was used for comparison between groups, and if not, the rank sum test was used for comparison between groups. The enumeration data were expressed by *n* (%), and the comparison between groups was performed by *χ*^2^ test or Fisher's exact test (*n* < 5). *P* < 0.05 was statistically significant.

## Results

### General data

According to the inclusion and exclusion criteria, a total of 85 patients with spontaneous isolated superior mesenteric artery dissection were included, including 49 patients with conservative treatment and 36 patients with endovascular treatment. There were 74 males (87.06%) and 11 females (12.94%), aged 41–87 years, with an average of (56.80 ± 8.25) years. The mean BMI was 22.42 ± 1.24 kg/m^2^, and the mean time from symptom onset to hospital admission was 9.42 ± 3.29 days. The average NRS score was 4.44 ± 1.44. In the past history, there were 24 patients (28.2%) with long-term smoking, 23 patients (27.1%) with hypertension, 9 patients with hyperlipidemia (10.6%), and 3 patients with diabetes (3.5%). All 68 patients (100%) presented with abdominal pain and discomfort on admission, 18 patients (21.2%) were accompanied by abdominal distension symptoms, 6 patients (7.1%) were accompanied by diarrhea symptoms, and 2 patients (2.4%) were accompanied by bloody stool symptoms. There were 13 cases (15.3%) with nausea and vomiting symptoms, 2 cases (2.4%) with chest pain symptoms, and 4 cases (4.7%) with back pain symptoms, as shown in Table 1. The CT imaging results showed that 5 (5.9%) patients had dissecting aneurysm formation. The average true lumen diameter was 3.91 ± 1.36 mm, the average SAA was 62.76 ± 7.46°, the average distance from the opening of the SMA to the beginning of the dissection was 21.21 ± 4.09 mm, and the average length of the dissection was 52.91 ± 4.60 mm. There were 30 cases of severe occlusion of true lumen stenosis (≥70%), 9 cases of true lumen were completely occluded, and the remaining. The true lumen was unobstructed or only mildly stenotic in 55 cases. According to the Yun classification, 12 patients (14.1%) were type I, 20 (23.5%) were type IIa, 30 (35.3%) were type IIb, and 23 (27.1%) were type III, as shown in Table 2.

**Table 1:** General information of patients.

Normal information	*n* (%)/x ± s
Age	56.80 ± 8.25
Gender	
Male	74 (87.1)
Female	11 (12.9)
BMI	22.42 ± 1.24
NRS score	4.44 ± 1.44
Time from symptom onset to hospital admission (days)	9.42 ± 3.29
Clinical manifestations	
Stomach ache	85 (100.0)
Bloating	18 (21.2)
Diarrhea	6 (7.1)
Bloody stools	2 (2.4)
Feel sick and vomit	13 (15.3)
Chest pain	2 (2.4)
Back pain	4 (4.7)
Past history	
Smoking history	24 (28.2)
Hypertension	23 (27.1)
Hyperlipidemia	9 (10.6)
Diabetes	3 (3.5)

BMI, body mass index; NRS, numerical rating scales.

**Table 2:** The imaging data of the patients.

Normal information	*n* (%)/x ± s
Imaging features	
Dissecting aneurysm	5 (5.9)
True lumen diameter	3.91 ± 1.36
Abdominal aorta—superior mesenteric artery angle	62.76 ± 7.46
SMA opening to start of interlayer (mm)	21.21 ± 4.09
Interlayer Length (mm)	52.91 ± 4.60
Yun type	
Type I	12 (14.1)
Type IIa	20 (23.5)
Type IIb	30 (35.3)
Type III	23 (27.1)

SMA, superior mesenteric artery.

### Relationship between treatment modality and clinicopathological factors in SISMAD patients

**Figure 2:**
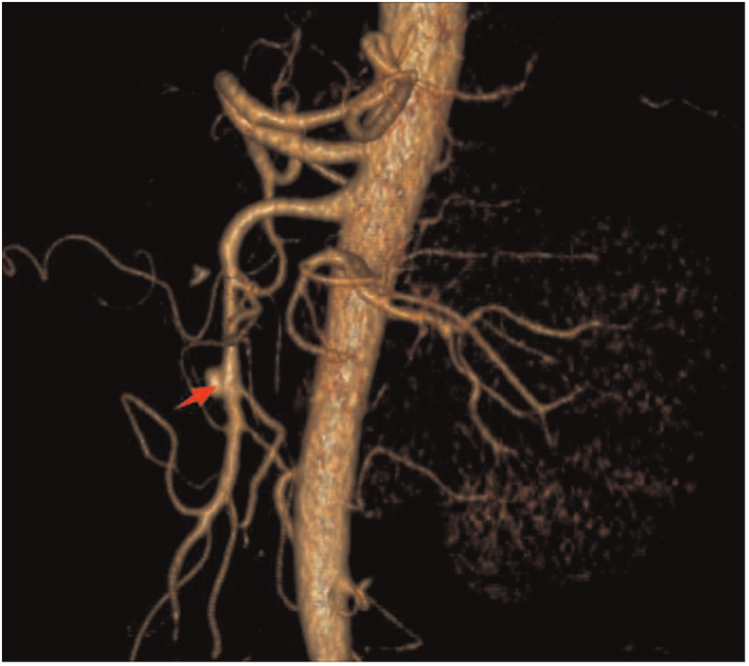
Comparison of true lumen stenosis rate between conservative treatment group and endoluminal treatment group.

In this study, 49 patients received conservative treatment and 36 patients received endovascular treatment. There was no significant difference in gender and BMI between conservative treatment and endovascular treatment (*P* = 0.278, *P* = 0.350). The clinical manifestations of both groups were mainly abdominal pain, with abdominal distension, diarrhea, bloody stool, nausea, and vomiting. There was no significant difference in clinical manifestations, such as chest pain and back pain (*P* = 0.738, *P* = 0.643, *P* = 0.220, *P* = 0.763, *P* = 0.825, *P* = 0.751), and past history, such as smoking history, hypertension, and high fat. There was no significant difference in blood sugar and diabetes mellitus (*P* = 0.570, *P* = 0.714, *P* = 0.562, *P* = 0.748); however, there were significant differences in age and preoperative NRS score between the two groups (*P* = 0.009). Regarding the imaging characteristics of the patients in this study, the most common Yun classification was type IIb in the conservative treatment group (21 cases, 42.86%); the most common Yun classification was type III in the endovascular treatment group (16 cases, 44.44%). There were significant differences between the conservative treatment group and the endovascular treatment group in the preoperative true lumen diameter, the distance from the opening of SAA, SMA to the beginning of the dissection, the length of the dissection, the true lumen stenosis rate, and the Yun classification (*P* < 0.001, *P* < 0.001, *P* < 0.001, *P* < 0.001, *P* < 0.001, *P* < 0.001, *P* < 0.001, *P* < 0.001, *P* < 0.001, *P* = 0.018), as shown in Table 3. The rate of true lumen stenosis in endovascular treatment was higher than that in conservative treatment group, as shown in Figure 2.

**Table 3:** Comparison of patients in the conservative treatment group and the endovascular treatment group.

Group	Conservative treatment (*n* = 49)	Endovascular therapy (*n* = 36)	*t*/Z/*χ*^2^	*P*-value
Age	58.78 ± 8.60	54.11 ± 7.01	2.667	0.009
Gender				
Male	41 (83.67)	33 (91.67)	1.177	0.278
Female	8 (16.33)	3 (8.33)		
BMI	22.53 ± 1.46	22.27 ± 0.87	0.940	0.350
NRS score	3.49 ± 0.94	5.72 ± 0.91	−10.960	<0.001
Clinical manifestations				
Stomach ache	49 (100)	36 (100)	—	—
Bloating	11 (22.45)	7 (19.44)	0.112	0.738
Diarrhea	4 (8.16)	2 (5.56)	0.215	0.643
Bloody stools	2 (4.08)	—	1.505	0.220
Feel sick and vomit	7 (14.29)	6 (16.67)	0.091	0.763
Chest pain	1 (2.04)	1 (2.56)	0.049	0.825
Low back pain	2 (4.08)	2 (5.56)	0.101	0.751
Past history				
Smoking history	15 (30.61)	9 (25.00)	0.323	0.570
Hypertension	14 (28.57)	9 (25.00)	0.134	0.714
Hyperlipidemia	6 (12.24)	3 (8.33)	0.335	0.562
Diabetes	2 (4.08)	1 (2.78)	0.104	0.748
Imaging features				
Dissecting aneurysm	3 (6.12)	2 (4.08)	0.012	0.913
True lumen diameter	4.94 ± 0.63	2.50 ± 0.61	17.944	<0.001
Abdominal aorta—superior mesenteric artery angle	59.99 ± 7.24	66.52 ± 6.04	−4.400	<0.001
SMA opening to start of interlayer (mm)	24.26 ± 1.77	17.07 ± 2.25	16.492	<0.001
Interlayer length (mm)	56.25 ± 2.41	48.376 ± 2.44	14.805	<0.001
True lumen stenosis rate (%)	50.05 ± 20.06	75.84 ± 13.33	−6.698	<0.001
Yun type			10.103	0.018
Type I	7 (14.29)	5 (13.89)		
Type IIa	14 (28.57)	6 (16.67)		
Type IIb	21 (42.86)	9 (25.00)		
Type III	7 (14.28)	16 (44.44)		

BMI, body mass index; NRS, numerical rating scales; SMA, superior mesenteric artery.

### Follow-up data

In the endoluminal treatment group, the preoperative and preoperative evaluation difference of NRS score was 3.61 ± 1.23, and the average difference before and after vascular true lumen diameter was 3.92 ± 0.97 mm. The differences were statistically significant (Z-test values were −6.282 and −6.397, *P* < 0.001; Table 4); in the conservative treatment group, the preoperative and preoperative evaluation difference of NRS score was 1.94 ± 0.63, and the average difference before and after the true lumen diameter of blood vessel was 1.14 ± 0.46 mm. Statistical significance (Z-test values were −5.298 and −5.297, *P* < 0.001; Table 4). The mean follow-up time of patients was 36.58 ± 25.03 months. The average total hospitalization cost of the conservative treatment group was 11,096.25 ± 1,769.16 Yuan, which was lower than that of the endovascular treatment group (36,594.12 ± 2,215.36 Yuan), and the difference was statistically significant (*P* < 0.001). The average hospitalization time of the conservative treatment group was 7.50 ± 0.56 days, shorter than the endovascular treatment group (8.80 ± 0.52 days), and the difference was statistically significant (*P* < 0.001). The follow-up complication rates of conservative treatment patients and endovascular treatment patients were 6.12% (3/49) and 11.11% (4/36), and the difference was not statistically significant (*P* > 0.05), as shown in Table 5. Among the 49 patients in the conservative treatment group, 1 patient was admitted to the hospital with abdominal pain as the chief complaint for 3 days and was given anticoagulation therapy. He died after the 39th day of admission. The remaining 47 patients were treated with conservative treatment, the symptoms of abdominal pain were gradually relieved, and there was no recurrence of abdominal pain and discomfort during the follow-up period; among the 36 patients in the endovascular treatment group, 1 patient underwent intestinal resection due to intestinal avascular necrosis on the 64th day. Serious and poor prognosis death; 1 patient died of acute myocardial infarction with mesenteric artery dissection, blood pressure control + antiplatelet, emergency cardiac intervention, and died 1 h after surgery; 1 patient died of renal failure with perirenal hemorrhagic shock after discharge. The remaining 33 cases underwent endoluminal stent implantation: 1 case was implanted with a bare EV3 stent and then a Bard covered stent was implanted, 1 case was implanted with a Bard covered stent, 3 cases were implanted with an EV3 bare stent Parallel spring embolization was implanted, 2 bare EV3 stents were implanted in 5 cases due to the extensive involvement of dissection, and 1 bare EV3 stent was implanted in the remaining 24 cases (Table 6). CTA was reviewed 6 months and 1 year after the operation, and the stent was unobstructed. Once case showed stent occlusion in the follow-up CTA 5 years after operation, and the symptoms improved after dual antibody treatment and was discharged from the hospital.

**Table 4:** Comparison of curative effect indexes before and after treatment in the two groups.

Group	Conservative treatment (*n* = 49)				Endovascular therapy (*n* = 36)			
	Preoperative	Postoperative	Z	*P*-value	Preoperative	Postoperative	Z	*P*-value
NRS score	3.49 ± 0.94	1.55 ± 0.65	−6.282	<0.001	5.72 ± 0.91	2.11 ± 0.79	−5.298	<0.001
true lumen diameter	4.94 ± 0.63	6.08 ± 0.67	−6.397	<0.001	2.50 ± 0.61	6.42 ± 0.84	−5.297	<0.001

NRS, numerical rating scales.

**Table 5:** Comparison of follow-up data of two groups of patients.

Group	Conservative treatment (*n* = 49)	Endovascular therapy (*n* = 36)	*t*/Z/*χ*2	*P-*value
Average hospital costs	11,096.25 ± 1769.16	36,594.12 ± 2215.36	14.805	<0.001
Average length of hospital stay	7.50 ± 0.56	8.80 ± 0.52	6.113	0.008
Complication	3 (6.12)	4 (11.11)	0.683	0.408
Ending			0.678	0.410
Survival	47 (95.92)	33 (91.67)		
Death	2 (4.08)	3 (8.33)		
Length of follow-up	33.26 ± 12.15	40.45 ± 12.93	0.890	0.312

**Table 6:** Surgical data of endovascular treatment group.

Group	Bare stent implantation (*n* = 30)	Bare stent implantation with spring embolization (*n* = 3)	Stent graft implantation (*n* = 2)
Treatment plan			
Single	25 (6.12)	3 (6.12)	2 (8.33)
Double	5 (6.12)	0	0
Result			0.678
Success	30 (100)	33 (100)	1 (50)
Failure	0	0	1 (50)
Secondary surgery	0	0	1 (50)

## Discussion

As a rare disease, SISMAD has a rising diagnostic positive rate with the continuous updating of imaging diagnostic techniques. However, its corresponding treatment strategy is still controversial, and its related risk factors and pathophysiological mechanisms have not yet been clarified. There is also a lack of unified diagnosis and treatment standards. Conservative treatment, as the first-line therapy recommended by the European Society of Vascular Surgery (ESVS) guidelines (7), mainly includes blood pressure control, fasting water, blood lipid lowering, antiplatelet, anticoagulation, etc. Conservative treatment has poor efficacy, combined with rupture of dissecting aneurysm, and consideration of intestinal ischemia. If intestinal ischemia causes complications related to intestinal necrosis, it should be converted to open surgery for necrotic bowel resection. In this study, 25 cases (69.44%) of the 36 patients in the endovascular treatment group had a disease course of 2 weeks or more and had received conservative treatment for a certain period of time. Endovascular therapy is expected to become the first-line therapy for SISMAD due to its advantages of less trauma and rapid recanalization of blood vessels (10). However, the current reports on endovascular treatment of SISMAD are limited due to the small number of cases (11). In this context, this study retrospectively analyzed the data of 85 SISMAD patients admitted to our hospital, including 49 cases of conservative treatment and 36 cases of endovascular treatment, and analyzed and discussed according to their corresponding curative effects.

In this study, male patients accounted for 87.1%, and the age was 56.80 ± 8.25 years. According to relevant literature reports, patients with SISMAD are mostly male, up to 67%–91%, and their age is mostly concentrated in 50–60 years old, and most of them are distributed in East Asia (China, Japan, etc.), considering geographical and ethnicity (12–14). Diabetes, hypertension, hyperlipidemia, and smoking have all been reported as risk factors for SISMAD (5). Also, in this study, there were 24 patients (28.2%) with long-term smoking, 23 patients (27.1%) with hypertension, 9 patients with hyperlipidemia (10.6%), and 3 patients with diabetes (3.5%). Among the subjects included in this study, abdominal pain was the main clinical manifestation in 100% of the cases, with abdominal distension in 21.2%, diarrhea in 7.1%, bloody stool in 2.4%, nausea and vomiting in 15.3%, chest pain in 2.4%, and back pain in 4.7%. The clinical symptoms of SISMAD lack specificity, and most patients present with sudden epigastric pain and discomfort. It is reported that the degree of SISMAD significantly correlated with the degree of true lumen stenosis and the length of dissection (15) and may be accompanied by nausea, vomiting. and other discomfort. Mild cases may have no symptoms, while severe cases may have corresponding peritoneal irritation signs or even cause intestinal necrosis. Therefore, for patients with unexplained abdominal pain (especially patients whose clinical symptoms and signs are inconsistent), special vigilance should be made for vascular-related diseases.

The patients included in this study were all diagnosed by enhanced CT or CTA, and the diagnostic rate was as high as 95%, which has become the preferred method for clinical diagnosis of SISMAD (16). According to the imaging results, the average SAA was 62.76 ± 7.46°, and the average distance from the SMA opening to the beginning of the dissection was 21.21 ± 4.09 mm. Relevant literature reports that the entrance of dissection is mostly located 10–30 mm away from the origin of SMA. In this area, the transition of SMA occurs from the relatively fixed posterior pancreas to the relatively unstable mesentery, and the blood vessels in this area are sheared due to the impact of blood flow. The force is higher than that of clinical blood vessels, and its related hemodynamic parameters are changed, which can easily lead to the occurrence of dissection (17, 18). Ying et al. (19) followed up 22 SISMAD patients and found that the length of dissection (OR = 2.132, 95% CI = 1.100–4.530, *P* = 0.025) and the degree of true lumen stenosis (OR = 3.250, 95% CI = 1.215–4.830, *P* = 0.032) were risk factors for vascular remodeling. Similarly, Min et al. (20) also pointed out that SAA is an independent risk factor for the incidence of ISMAD, showing a significant positive correlation, and the risk of ISMAD gradually increases with the increase of SAA. In Yun's classification, type III was the most common in the endovascular treatment group (44.44%), followed by type II (41.67%). Xu et al. (21) pointed out that most patients with type I do not need clinical intervention, while endovascular treatment is mostly suitable for patients with type II and type III. In this study, there were more type III than type II patients. Considering that type III patients had more severe true lumen stenosis and more obvious intestinal ischemia than type II patients, and type I patients were less stenotic by false lumen compression, they could accept endovascular treatment. In the conservative treatment group, type IIb was the most common (42.86%), and type II accounted for 71.43%. Excluding the effect of the diameter of the false lumen, because the risk of false lumen rupture is small, and the thrombus in the false lumen is usually absorbed gradually and the stenotic true lumen is gradually remodeled, conservative treatment is preferred for type II patients (22).

Since Leung et al. (23) first applied endovascular surgery to the treatment of SISMAD in 2000, endovascular surgery for SISMAD has developed rapidly. Approximately 20% of SISMAD patients receive endovascular therapy (7). In the endovascular treatment group, the success rate of interventional surgery was as high as 86.11% (31/36), of which one case was terminated because the patient was in poor physical condition and could not tolerate the operation, and four cases failed because the guide wire could not enter the true cavity. Whether the guide wire can pass through the true lumen depends on the characteristics of the SISMAD lesions (for lesions with thrombosis in the true lumen or occlusion of the true lumen due to excessive false lumen, the surgical success rate is often low). According to relevant literature reports, the success rate of endovascular treatment is 60%–100% (24). Dong et al. (25) summarized the experience of eight cases of failure of endoluminal therapy and showed that the guide wire failed to be selected into the true lumen. Endovascular treatment is mainly based on stent implantation (mainly bare metal stent) as the most common method. Other methods include balloon dilation, spring embolization, catheter thrombolysis, etc. (26, 27), which are formulated according to the patient's condition and imaging results and the corresponding treatment plan. At present, it is believed that stent implantation can provide a very good short-term effect for the revascularization of the superior mesenteric artery (28), mainly including bare stents and covered stents. However, it is still difficult to choose a covered stent or a bare stent for the treatment of superior mesenteric artery dissection since there is controversy. The advantage of the bare stent is that it has a protective effect on the branches of the superior mesenteric artery, and it is a good choice for the treatment of dissection very close to the branch; the disadvantage may be that the bare stent is prone to the risk of endoleak, and the bare stent was placed in this study. There were no endoleaks, so bare stents remain one of the stent options of choice for the treatment of superior mesenteric artery dissection. The advantage of the covered stent is that it can seal the rupture of the dissection and the accompanying superior mesenteric aneurysm (29). When the angle between the abdominal aorta and the superior mesenteric artery is small, it is difficult for the stent graft to enter the superior mesenteric artery. In this study, two patients were implanted with covered stents, and one of them was implanted with a covered stent combined with a bare stent (due to the large rupture, the false lumen blood supply was still abundant after bare stent implantation; therefore, a covered stent was added). Hence, when choosing stent treatment, bare stents, covered stents, or a combination of the two should be selected according to the specific circumstances of the angiography.

In the conservative treatment group of this study, two patients died, of which one died after the 39th day of admission and one died 10 h after admission; the general condition of the remaining 47 patients was good, and no patient died during the follow-up period. It is generally believed that conservative treatment is mainly suitable for patients with stable disease, no signs of peritoneal irritation or intestinal necrosis, SMA true lumen stenosis but well-compensated collateral vessels, aneurysm diameter <2 cm, and no dissecting aneurysm rupture or signs of rupture; some patients with poor physical condition who cannot tolerate interventional and open surgery finally chose conservative treatment (30). Most symptomatic SISMAD patients have good conservative treatment effects. Garrett (31) showed that the success rate of conservative treatment was 86.6% through clinical research results. Among the 36 patients in the endovascular treatment group, two patients died, including one patient with acute myocardial infarction and 1 h after emergency cardiac interventional surgery, one patient due to renal failure with perirenal hemorrhagic shock, and one patient due to intestinal defect. The patients with blood necrosis had severe prognosis and died on the 64th day after enterectomy. The general condition of the remaining 33 patients was good. The CTA of 1 patient showed stent occlusion at 5 years after operation, and the symptoms improved after dual antibody treatment. Long-term complications of endoluminal therapy include in-stent restenosis, stent occlusion, and stent thrombosis (32). A meta-analysis from 2018 suggested (26) that only 2 of 97 patients with endovascularly treated SISMAD underwent secondary intervention due to stent restenosis and vasodilation. For the included patient with stent occlusion, we considered that the patient was generally in good condition and had no clinical symptoms, so endovascular treatment was not performed.

This study has certain limitations. On the one hand, this study is a retrospective study, with a small number of cases from a single center, and the comparison of the efficacy of conservative treatment and endovascular treatment of SISMAD needs stronger evidence to support; on the other hand, the follow-up time is up to 86 months. Longer follow-up time is needed to compare the effects of different surgical methods.

In conclusion, the results of this study show that conservative treatment is effective for most symptomatic SISMAD patients, but there are still some patients whose symptoms persist and need endoluminal therapy. Endovascular treatment is preferred; in the long-term, most patients maintain patency of SMA after surgery, with low symptom recurrence and low complication rates. Both conservative treatment and endovascular treatment are effective treatment methods for SISMAD patients.

## Data Availability

The original contributions presented in the study are included in the article/Supplementary Material, further inquiries can be directed to the corresponding author.
